# An Unusual Granular Cell Tumour of the Buttock and a Review of Granular Cell Tumours

**DOI:** 10.1155/2013/109308

**Published:** 2013-08-26

**Authors:** Sharad P. Paul, Vladimir Osipov

**Affiliations:** ^1^Skin Surgery Clinic, 271A Blockhouse Bay Road, Auckland 0600, New Zealand; ^2^Department of Skin Cancer, University of Queensland, Australia; ^3^Faculty of Surgery, University of Auckland, New Zealand; ^4^QML Pathology, Townsville, QLD, Australia

## Abstract

Granular cell tumours, first described by Abrikossoff in 1926, are known to occur in skin, connective tissue, breast, gastrointestinal and genital tracts. While they are rare, they are more common in people of African descent and show a slight female preponderance, usually presenting as solitary and painless masses. Less than 10% of occurrences are multiple, and fewer than 3% of tumours behave in a malignant fashion. The mean age, at presentation, is 40–60 years. We report a case of granular cell tumour in a young white male presenting with a painful soft tissue tumour in his buttock. The presentation is unusual because of the age, patient demographic, body site, and clinical presentation. The clinical and histological aspects are reviewed in the context of this clinical case and the associated literature.

## 1. Background

Granular cell tumours were first described by Abrikossoff in 1926 and are known to occur in skin, connective tissue, breast tissue, and gastrointestinal and genital tracts—with the head and neck being the most common regions. The tongue is the most common site in the head and neck [1]. Granular cell tumours are rare; some authors have suggested that they make up around 0.5% of all soft tissue tumours [2]. Frequent locations are the tongue (40%), breast (15%), respiratory tract (10%), and oesophagus (2%) [3]. The tumours can be multicentric (5% to 14% of cases) [3]. These tumours have a higher incidence amongst women and a greater prevalence amongst people of African descent. There has been one case report of a mother and son, both of whom presented in childhood with multiple granular cell tumours [4]. While the origins of granular cell tumours are often debated, Abrikossoff originally postulated a myogenic origin and termed this a “myoblastoma.” These tumours are now considered to be neoplasms of neural origin, as evidenced by immunohistochemical studies [5]. Diffuse S-100 positivity is present in nearly every case. S-100 is a calcium binding protein expressed in nerve tissue, melanocytes, adipocytes, and myoepithelial cells. Dermal nonneural granular cell tumours may be a different entity [6]. It is difficult to make a diagnosis of malignancy in these tumours based on the histological appearance. Tumours that do metastasize tend to show cellular pleomorphism, mitotic activity, and spindling. Size greater than 5 cm, rapid growth rate, or invasion of adjacent structures is more likely to suggest malignancy [7]. Most granular cell tumours are benign, with a self-limiting growth pattern. When they metastasize, the most common sites are regional lymph nodes, lungs, or bones [8]. Granular cell tumours are rare on the trunk and usually present as a solitary, painless mass, with the patient usually noticing a lump [9].

The case we are reporting is that of a young white male, aged 27, who presented with a two-month history of a 2 cm mass in his buttock, which was preventing him from sitting down, due to pain. Our initial clinical impression of this fibrofatty mass was of a well-circumscribed lipoma or neurofibroma, and the differential diagnosis included a cyst. The pain and tenderness to touch were attributed to pressure effects on his sciatic nerve. Given that this was a young male patient presenting with a painful dermal/subcutaneous mass, we did not consider granular cell tumour as part of our differential diagnosis until histopathological examination.

## 2. Case History

A 27-year-old white male was referred to our centre, by his GP, with a lump in the patient's right buttock. The mass was 2 cm in diameter and was felt to be a lipoma clinically. The patient himself was not aware of the lump and had visited his GP only because every time he sat down he felt pain over his buttock region, which radiated down his leg. This symptom was easily reproducible and prevented the patient from sitting down on hard surfaces. The patient was otherwise well, with no other medical conditions. There was no family history of any malignancy or cutaneous masses or lipomata.

On examination, we felt a well-localized, approximately 2 cm soft tissue mass, which was clinically located in the deep dermis or in the subcutaneous fat. There was no attachment to muscle and no overlying skin changes. Our differential diagnoses included lipoma, neurofibroma, or cyst. Given that the lesion was well localized, not greater than 2 cm and not adherent to muscle or deep fascia, we proceeded to excise the lesion under local anaesthesia, without imaging.

During the operation, the lesion seemed well localized and intraoperatively resembled a sebaceous cyst or pilomatrixoma. 

Histological reports are detailed below. A compete skin and lymph node examination revealed no other abnormalities. After reviewing the histopathology, this patient was managed with a wide local excision with 1 cm margins.

## 3. Histopathological Examination

**Figure 1:**
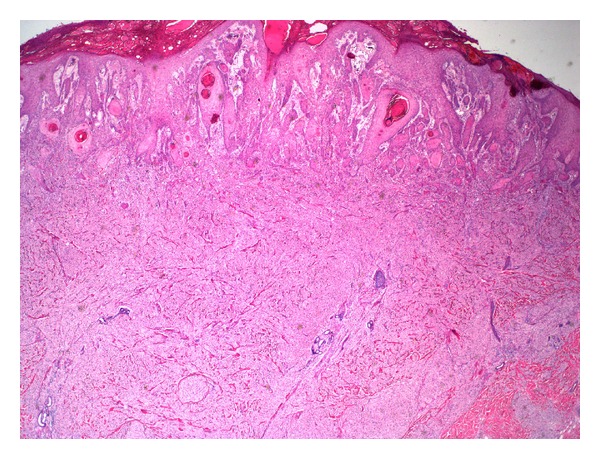
H&E, 20x magnification.

**Figure 2:**
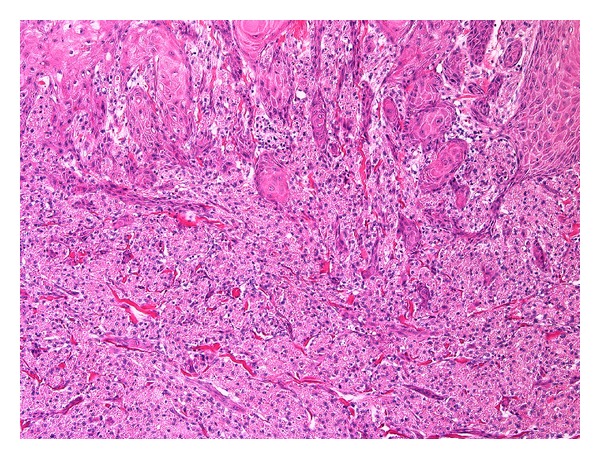
H&E, 100x magnification.

The tumour was well circumscribed, spanned the entire dermis, and showed broad interface with the underlying adipose tissue. The interface with the epidermis was quite irregular, with prominent epidermal pseudoepitheliomatous hyperplasia (Figures 1 and 2).

**Figure 3:**
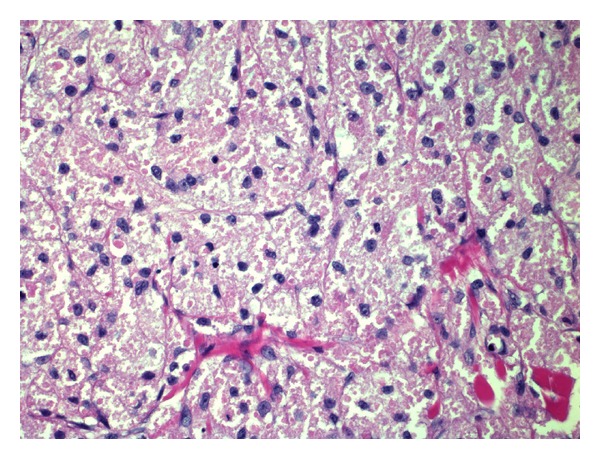
H&E, 400x magnification.

Histologically, the tumour cells were quite monomorphous, with small round nuclei and abundant granular eosinophilic cytoplasm (Figure 3). The tumour cells were diffusely positive with S-100 immunohistochemical stain. There were no features of malignancy, such as nuclear pleomorphism, necrosis, spindling, or mitotic activity.

## 4. Discussion

Granular cell tumours are uncommon and when they occur, they are most common in the head and neck. They present as painless masses. Surgical excision is the treatment of choice. The recurrence rate for granular cell tumours has been reported at 2%, when local wide excision has been undertaken [10]. Most granular cell tumours can be easily managed by wide local excision; in cosmetically sensitive areas, where tissue preservation is paramount, Mohs Micrographic Surgery has been used [11]. 

Histologically the tumour presented no diagnostic difficulty. However if the tumour was sampled superficially, the irregular interface with the overlying epidermis would create a well-known diagnostic pitfall. With superficial biopsy, one can see how easily a diagnosis of invasive, well-differentiated, squamous cell carcinoma can be made. This can lead to a potentially harmful, unnecessary surgery, especially when the lesion is present in the tongue. 

Our patient presentation was unusual, given that it was the symptomatic nature of the lesion that led to the diagnosis—with the patient being unable to sit due to buttock pain—which resulted in the initial referral; the impression, prior to surgery, of a neuroma; during surgery, a cyst or pilomatrixoma. A survey of buttock tumours had suggested that when pain is present it is usually due to cyst formation in an old haematoma, and pain along the course of the sciatic nerve, and its branches, was present in 40 percent of the cases [12].

Malignant behaviour is rare and is seen in up to 2% of the cases. Most of the malignant tumours arise in the thigh, while malignant granular cell tumours of the head and neck are very rare [13]. The malignant granular cell tumours are more common in African American women. Age of the patients with malignant tumours is the same as for benign cases, that is, 30–50 years. The treatment of choice is complete wide local excision, as was performed on our patient. There are no clinical or histopathologic features to suggest that the tumour we present will behave in a malignant fashion.

The tumour we present was excised completely, as incomplete excision may produce recurrences in 21–50% of the cases [14]. 

This case report suggests that granular cell tumours must be considered in the differential diagnosis of painful buttock tumours.
